# Meeting the needs of the resident trainee during an elective subspecialty rotation

**DOI:** 10.5116/ijme.56f5.c7ec

**Published:** 2016-04-10

**Authors:** Andrew Hale, Rebecca Glassman, David Fessler, Kenneth J. Mukamal, Wendy Stead

**Affiliations:** 1 Division of Infectious Disease at Beth Israel Deaconess Medical Center and Harvard Medical School, USA; 2 Division of General Medicine and Primary Care at Beth Israel Deaconess Medical Center and Harvard Medical School, USA

**Keywords:** Residency curricula, subspecialty electives, faculty development, internal medicine resident education

## Abstract

**Objective:**

To examine and compare perceptions between resident-trainees and faculty-educators on goals and reasons why resident trainees choose certain subspecialty elective rotations.

**Methods:**

In June 2013 residents and faculty-educators at a large tertiary care academic medical center were surveyed regarding perceived resident goals for subspecialty electives. Each group was sent a different electronic survey of parallel questions assessing agreement on an ordered scale with statements about which factors impacted resident choice.

**Results:**

The survey was sent to 154 residents and had 75 (49%) respondents, as well as 20 faculty-educators with 12 (60%) respondents. Residents and faculty did not differ in their responses that electives were chosen to fill perceived knowledge gaps (exact Cochran-Armitage p = .51). However, educators and residents significantly varied in the degree to which they thought resident choice was based on networking within the field (exact Cochran-Armitage p = .01), auditioning for fellowship (exact Cochran-Armitage p < .01), or exploring career options (exact Cochran-Armitage p = .01), with educators overestimating the degree to which these impacted resident choice.

**Conclusions:**

Resident trainees and faculty educators agree that subspecialty electives are most frequently chosen in order to meet resident educational goals, highlighting the importance of developing and delivering high quality subspecialty curricular content for the internal medicine resident learner during electives. Many residents choose electives for career development reasons, but faculty educators overestimate this motivation.

## Introduction

During a typical three years of residency training, internal medicine residents in the United States choose to participate in subspecialty electives for many different reasons. Residents may choose electives to pursue career interests, perhaps with the hope of networking or securing letters of recommendation.^1^ They may choose electives of high educational value to fill their own perceived knowledge gaps.^2^ In addition, certain elective rotations may be required by the residency program. As a result of varying goals and lack of formal curricular development, subspecialties themselves may not be prepared to focus their teaching specifically to the resident learner. The resident learner on a subspecialty elective is often taken on a brief tour of that specialty, learning primarily from participation in rounds and ambulatory clinics, but often with little structured curriculum focusing on core concepts within that subspecialty.  The teaching on a subspecialty consultative service at an academic teaching hospital often focuses on clinical fellows, who have completed residency. Extensive time is spent ensuring adequate exposure to specific clinical issues, designing conferences and didactic sessions to maximize fellow learning and address key curricular content distributed over the course of one or more years. However, the needs of learners at the fellow level do not always overlap with the needs of the internal medicine (IM) resident learner, and the limited time on service for the resident learner may impact their exposure to the fellows’ structured curriculum.

A handful of small studies of subspecialty electives suggest that carefully designed subspecialty curricula for residents can lead to improvements in standardized test scores and affect residents’ choices of subspecialty fellowship.^3^^-^^7^ There is otherwise little guidance regarding optimizing the subspecialty consult elective experience for the internal medicine resident learner in medical education literature. The Accreditation Council for Graduate Medical Education (ACGME) provides limited advice on these matters, tasking program directors with identifying board certified faculty to serve as “subspecialty education coordinators [SSECs], accountable for coordination of the residents’ subspecialty educational experiences in order to accomplish goals and objectives in the subspecialty.”^8^ However, beyond listing “the core content of general internal medicine which includes the internal medicine subspecialties” as a requirement of the medical knowledge competency, the ACGME program requirements for graduate medical education in internal medicine leave the building blocks for subspecialty curricular development in the hands of residency programs and SSECs. ACGME requirements for the subspecialties themselves offer even less guidance regarding responsibilities toward IM resident rotators except to state that “the presence of other learners (including, but not limited to, residents from other specialties, subspecialty fellows…) must not interfere with the appointed fellows’ education.”^9^

Given the amount of time the average resident training in internal medicine spends doing subspecialty electives during three years of training, in addition to the potential impact of these subspecialty exposures on future career choices, further evaluation of residents’ goals to guide subspecialty elective curricular development is needed. In this study, we conducted surveys of both residents and faculty-educators to elucidate residents’ primary goals for subspecialty elective time and to explore discrepancies that may exist between resident and faculty-educator objectives for subspecialty rotations. We hypothesized that residents and faculty-educators may have different perceptions regarding what residents want to gain from subspecialty elective time, with the hope that identifying discrepancies between resident and faculty-educator goals could help to inform future curricular development for subspecialty elective rotations.  

## Methods

### Location and participants

We conducted a survey of internal medicine residents, internal medicine program faculty leadership, and Subspecialty Education Coordinators (SSECs). The study occurred at Beth Israel Deaconess Medical Center (BIDMC) in June 2013. BIDMC is a 649-bed tertiary care academic center affiliated with Harvard Medical School. The BIDMC Department of Medicine had 154 residents (post-graduate year 1, 2 and 3 physicians-in-training) at the time of the study. The Department of Medicine at BIDMC has a full complement of medical subspecialty consult services through which residents may choose to rotate. There are a total of 15 SSECs and 5 residency program directors, and thus 20 faculty-educators were eligible to be surveyed. Subspecialty rotations average 1-2 weeks in length. At BIDMC, an IM resident will typically spend 8-20 weeks (depending on research time and nontraditional electives such as global health rotations) on subspecialty elective services during three years of training.

### Study design and survey

Two electronic surveys were created and distributed electronically; one was directed at all internal medicine residents, the other at faculty-educators (residency program directors and Subspecialty Education Coordinators). The resident survey was sent to 154 residents (all IM residents at BIDMC) at the end of the curricular year (June 2013) to maximize exposure to elective rotations. The survey consisted of ten questions aimed at understanding why residents chose a particular subspecialty elective, and what goals they had for the subspecialty elective experience. The faculty-educator survey consisted of ten parallel questions to determine overlap and discrepancies between resident and faculty-educator perceptions. Participants responded using a 5-point Likert scale (strongly agree, agree, neutral, disagree, and strongly disagree). Participation for both groups was voluntary and anonymous, and no incentives were provided for either group. The study was reviewed by the Beth Israel Deaconess Medical Center Institutional Review Board, which determined that the study did not meet requirements for Human Subjects Research.

### Statistical analysis

Statistical analysis was performed using SAS software (version 9.3, Cary, NC). To compare ordinal responses (on the 5-point Likert scale) between residents and faculty-educators, we used an exact Cochran-Armitage test for trend for all comparisons (PROC FREQ, EXACT STATEMENT).^10^ P value of 0.05 was used to determine significance for all tests.  

## Results

The resident survey was completed by 75 of 154 residents (49% response rate). Of these, 26 were PGY1 (of 61 total PGY1 residents; 43% PGY1 response rate), 26 PGY2 (of 46 total PGY2 residents; 57% PGY2 response rate), and 23 PGY3 (of 47 total PGY3 resident; 49% PGY3 response rate). The faculty-educator survey was sent to 20 eligible faculty-educators, of which there were 12 respondents (60% of eligible pool). The intended career path identified by resident respondents showed 34% intending to pursue careers in general medicine (primary care or hospital medicine), 18% hematology-oncology, 13% pulmonary & critical care, and 12% cardiology; the remainder of subspecialties accounted for < 10% each. Of the 12 faculty-educators who responded, 9 were SSECs and 3 were IM residency program directors.

Residents and faculty-educators did not significantly differ in their perceptions that a primary reason residents

**Figure 1:**
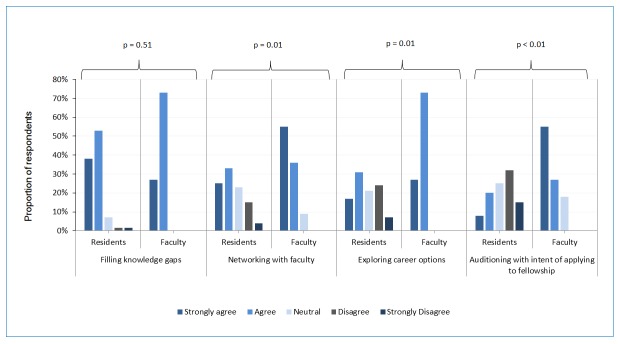
Survey at Beth Israel Deaconess Medical Center in June 2013 of internal medicine resident and faculty-educator perceptions on what factors impact the residents’ choice of subspecialty electives

choose a specific subspecialty elective is to fill perceived knowledge gaps (91% and 100% agreed, respectively. Figure 1, (exact Cochran-Armitage test of trend, N=87, Z= 0.66, p = .51)). However, residents and faculty-educators significantly varied in the degree to which they thought resident choice was based on networking within the field (58 and 91% agreed this was important, respectively (exact Cochran-Armitage test of trend, N=87, Z= -2.61, p = .01)), auditioning for fellowship within that department (28 and 82% agreed, respectively (exact Cochran-Armitage test of trend, N=87, Z= -4.27, p < .01)) and exploring career options (48 and 100% agreed, respectively (exact Cochran-Armitage test of trend, N=87, Z=-2.50, p = .01)). The groups did not differ in their assessment that perceived easier workload was infrequently a factor in choice of elective (37% of the time in both groups (exact Cochran-Armitage test of trend, N=87, Z= -0.46, p = .76).  Figure 1 shows the comparison between resident and faculty-educator responses. For each question, each groups’ response on the 5-point Likert scale is visually represented, with p values reported above shown for comparison.

## Discussion

In US residency training programs in internal medicine, residents’ learning environment traditionally includes core rotations on general medicine inpatient wards, intensive care units, and primary care clinics. Additionally, subspecialty electives comprise a significant portion of the training experience in most internal medicine residency training programs. While there is significant literature published on resident-focused teaching and curriculum in the core rotations,^11^^-^^18^ less has focused on what exactly residents should get from their time on subspecialty electives,^19^ and none has previously explored trainee and faculty-educator goals for this time. Our study sheds light on IM resident goals for subspecialty elective time, as well as potential misperceptions by faculty-educators regarding trainee motivations for choosing certain subspecialty electives. We found that residents choose particular subspecialty electives primarily to learn new material and fill perceived knowledge gaps, and faculty-educators agreed that this was the anticipated goal. This shared goal suggests SSECs should develop more formalized curricula based on needs assessments of the programs’ trainees.^20^ Additionally, our study showed that an easier workload was infrequently felt to drive subspecialty elective choice, suggesting that residents value the learning gained from these electives and do not simply seek respite from the demands of their regular schedule. Interestingly, faculty-educators overestimated the emphasis residents put into choosing electives based on networking within that field, auditioning for fellowship, or exploring career options. Although these are important aspects of subspecialty electives, the desire of residents to acquire new medical knowledge stands out strikingly in the responses we received.

As many residents pursue careers in general internal medicine (34% in our study), and therefore may not receive the same exposure to experts in subspecialties, it is important that trainees develop an adequately comprehensive subspecialty knowledge-base during their residency training. Brief though it may be, subspecialty elective time for residents is a valuable opportunity to learn core material and should therefore be an educational priority.

Although faculty-educators overestimated resident interest in the career development aspects of subspecialty elective rotations, approximately half of IM residents nonetheless reported choosing subspecialty electives to network within the field and explore career options.  These data suggest that subspecialty electives continue to fulfill career development interests for a substantial minority of residents and attempts by SSECs to identify and mentor residents interested in their particular subspecialty fields may be worthwhile, a finding that would likely be particularly welcome by medical subspecialties that have suffered from waning applicant numbers in recent years.^21^^,^^22^  

Our study has several limitations. It was conducted at a single center and represents only a snapshot in time. Our study was limited to an academic medical center population, and thus may be less generalizable to community-based settings. The surveyed group was fairly small and survey response rate was relatively low (49% for residents and 60% for faculty educators); however, the difference between our groups was large enough to attain statistical significance for many of the variables measured. There may have been bias among those who answered our surveys and those who did not for which we cannot control. Additional research should be conducted with larger representative samples of faculty and resident trainees at other academic medical centers to see if these results are reproducible.

## Conclusions

Internal medicine residents spend a significant amount of their training on subspecialty electives. Despite this, previous studies have not explored resident goals for this time. Our hypothesis that residents and faculty-educators may have different perceptions regarding what residents want to gain from subspecialty elective time was confirmed. Our study suggests that acquisition of knowledge is residents’ primary reason for choosing a subspecialty elective and faculty-educators should prepare to meet that expectation with well-developed curricula. Many residents identify career exploration and networking with faculty as additional reasons to choose a subspecialty elective and faculty-educators should consider developing specialty-specific resources to meet this need during subspecialty elective time.

### Conflict of Interest

The authors declare that they have no conflict of interest.
